# Mass Spectrometry-Based System for Identifying and Typing Norovirus Major Capsid Protein VP1

**DOI:** 10.3390/v13112332

**Published:** 2021-11-22

**Authors:** Pei-Yu Chu, Hui-Wen Huang, Michittra Boonchan, Yu-Chang Tyan, Kevin Leroy Louis, Kun-Mu Lee, Kazushi Motomura, Liang-Yin Ke

**Affiliations:** 1 Department of Medical Laboratory Science and Biotechnology, College of Health Sciences, Kaohsiung Medical University, Kaohsiung 807, Taiwan; peiyuchu@kmu.edu.tw (P.-Y.C.); kev.l.louis05@gmail.com (K.L.L.); 2 Department of Laboratory Medicine, Kaohsiung Medical University Hospital, Kaohsiung 807, Taiwan; leekm1914@gmail.com; 3 Department of Anesthesiology, Kaohsiung Chang Gung Memorial Hospital and Chang Gung University College of Medicine, Kaohsiung 833, Taiwan; july25@cgmh.org.tw; 4 Thailand-Japan Research Collaboration Center on Emerging and Re-emerging Infections, Nonthaburi 11004, Thailand; michittra@hotmail.com; 5 Department of Medical Imaging and Radiological Sciences, Kaohsiung Medical University, Kaohsiung 807, Taiwan; yctyan@kmu.edu.tw; 6 Division of Public Health, Osaka Institute of Public Health, Osaka 543-0026, Japan; 7 Center for Lipid Biosciences, Department of Medical Research, Kaohsiung Medical University Hospital, Kaohsiung 807, Taiwan

**Keywords:** Norovirus, virus-like particle (VLP), M-class ultra-performance liquid chromatography system, data independent mass spectrometry (MS^E^), VP1 protein, P2-P1b region, genotyping

## Abstract

Norovirus-associated diseases are the most common foodborne illnesses worldwide. Polymerase chain reaction-based methods are the primary diagnostics for clinical samples; however, the high mutation rate of norovirus makes viral amplification and genotyping challenging. Technological advances in mass spectrometry (MS) make it a promising tool for identifying disease markers. Besides, the superior sensitivity of MS and proteomic approaches may enable the detection of all variants. Thus, this study aimed to establish an MS-based system for identifying and typing norovirus. We constructed three plasmids containing the major capsid protein VP1 of the norovirus GII.4 2006b, 2006a, and 2009a strains to produce virus-like particles for use as standards. Digested peptide signals were collected using a nano-flow ultra-performance liquid chromatography mass spectrometry (nano-UPLC/MS^E^) system, and analyzed by ProteinLynx Global SERVER and TREE-PUZZLE software. Results revealed that the LC/MS^E^ system had an excellent coverage rate: the system detected more than 94% of amino acids of 3.61 femtomole norovirus VP1 structural protein. In the likelihood-mapping analysis, the proportions of unresolved quartets were 2.9% and 4.9% in the VP1 and S domains, respectively, which is superior to the 15.1% unresolved quartets in current PCR-based methodology. In summary, the use of LC/MS^E^ may efficiently monitor genotypes, and sensitively detect structural and functional mutations of noroviruses.

## 1. Introduction

Human norovirus (HuNoV) is the leading cause of acute gastroenteritis (AGE) worldwide. Symptoms of infection include intense vomiting, nausea, watery diarrhea, abdominal cramping, and fever [1,2]. Young children, old age, and immuno-compromised status are associated with increased morbidity and mortality [3]. The HuNov spreads quickly in crowded environments, owing to its low infectious dose (<10^2^ viral particles), prolonged asymptomatic shedding, environmental stability, and strain diversity [4]. Of the approximately 685 million AGE cases reported annually, one-third are children younger than 5 years, and 210,000 are fatal [3,5]. Based on a recent phylogenetic analysis of the *VP1* gene and partial *RNA-dependent RNA polymerase* gene, norovirus is classified into 10 genogroups (GI-GX) and 49 genotypes. Most HuNoVs belong to genogroups GI and GII. The GII genotype 4 (GII.4) accounts for over 80% of AGE outbreaks worldwide [6].

Noroviruses are small (27–35 nm in diameter), naked, single-strand RNA viruses classified as genus *Norovirus* of the family *Caliciviridae* [7]. The RNA genome of HuNoV consists of three open reading frames (ORF 1–3). Among them, ORF2 encodes the basic building unit of viral capsid protein VP1 (58–60 kDa) [8]. The VP1 folds into two structurally and functionally independent domains: a conserved shell (S) domain that forms the icosahedral scaffold, and a variable protruding (P) domain for dimeric spikes extending from the shell. The P domain can be further sequentially subdivided into P1 subdomain 1 (P1-1), P2, and P1 subdomain 2 (P1-2). Self-assembly of 180 VP1 copies creates a virus-like particle (VLP) with a structure similar to that of a native virion, but with no infectivity [9]. Since VP1 is the major antigenicity determinant and the most abundant protein in the viral particle, VP1 is a potential target for identifying HuNoV infections.

The hypervariable P2 subdomain exposed on the top of the P domain is the main immune epitope. It participates in receptor binding, and is associated with immune escape [10]. Histo-blood group antigens (HBGAs) expressed on red blood cells and gastroduodenal epithelium are the cellular receptors for HuNoVs [11]. Due to polymorphism of HBGAs, and variation in P2 subdomains of HuNoVs, receptor-ligand interactions are group- and strain-specific [12]. A P2 subdomain generally has two HBGA-binding sites: a conserved central binding pocket at positions 343–345 in GII.4 HuNoV, and a variable surrounding region at positions 393–395 [13,14]. As mutations of these amino acids alter HBGA binding, new variants emerge every 3–7 years [15]. Consequently, detecting VP1 sequence variation is critical for the identification and surveillance of HuNoVs. For this purpose, a sensitive, high-throughput detection and typing system is mandatory.

Although 3-dimensional Caco-2 culture cells and stem cell-derived enteroids have been successfully used to isolate norovirus, they are still impractical for use in clinical laboratories [16,17]. Norovirus detection assays are still based on molecular diagnostics, either by traditional reverse transcription-polymerase chain reaction (RT-PCR) or real-time PCR [18,19]. However, the efficacy of primer-dependent methods is limited by the multiple genotypes and high mutation rate of HuNoVs. Alternatively, mass spectrometry (MS) technology has rapidly advanced this decade, and may be capable of detecting viral peptides with unprecedented sensitivity and specificity [20,21,22]. Notably, MS can also identify post-translational modification (PTM) of viral proteins [23]. Mass spectrometric analysis has become a promising tool for disease marker identification [24,25]. Thus, we aimed to set up MS-based system for identification and typing norovirus. Since PTM alters protein conformation and function, we analyzed the key functional sites and modifications of VP1 protein.

## 2. Materials and Methods

### 2.1. Study Design and Norovirus VP1-cDNA Preparation

As GII.4 2006b was the major leading cause of the strain during 2006–2009–world, virus strains GII.4 2006b, 2006a and 2009a (GII.4/Aichi3/2006/JPm UniProtKB: B5BTN4; GII.4/Saga5/2006/JP, UniProtKB: B5BTS0; and Hu/GII.4/Iwate4/2009/JP, UniProtKB: A0A0K2SRW5, respectively. All available online: https://www.uniprot.org/uniprot/, accessed on 11 October 2021) from our previous isolates were chosen to construct virus-like particles (VLPs) [26]. Briefly, RNA was isolated from stool samples from patients in Japan. RNA was extracted, and reverse transcription-polymerase chain reaction (RT-PCR) was analyzed. Positive samples were genotyped using sequencing. VLPs of three strains were produced at the Thailand-Japan Research Collaboration Center on Emerging and Re-emerging Infections—the materials and methods are listed in Section 2.2. At the Center for Lipid Biosciences of Kaohsiung Medical University Hospital, we further analyzed the amino acid sequences and modifications of VLPs by mass spectrometry.

### 2.2. The VP1 Protein Expression and Purification

Viral RNAs were extracted using a commercial kit (QIAgen, Hilden, Germany). The cDNA was obtained by reverse transcription of three virus strains using the primer Tx30 (5′- GACTAGTTCTAGATCGCGAGCGGCC-GCCC(T)_30_-3′) and SuperScriptTM III reverse transcriptase (Life Technologies, Carlsbad, CA, USA). Then, we amplified the entire ORF2 and ORF3 by PCR, using primers ORF2-1F (sense: 5′-CTATAAATAGATCTTGGTACCATGAAGATGGCGTCCAATGACCG-3′) and Tx30 (antisense). The 2484-bp PCR products were treated with Bgl II (Merck KGaA, Darmstadt, Germany) and NotI (New England Biolabs, MA, USA) restriction enzymes, and ligated into the pVL1392 vector (Invitrogen Life Technologies, Waltham, MA, USA). The plasmid was amplified by E. coli DH5α competent cells (Thermo Fisher Scientific, Waltham, MA, USA), then transfected into Sf9 insect cells (ATCC biotechnology, Manassas, VA, USA). The supernatant (50.0 mL) from infected cells was purified. The VLPs were purified by ultracentrifugation through a 30% (wt/vol) sucrose cushion, followed by cesium chloride (CsCl; Merck KGaA, Darmstadt, Germany) density gradient. The VLPs were then banded by CsCl gradient ultra-centrifugation at 36,000 rpm for 20 h. A total of 330 μL supernatant was fractioned for 10 tubes from the top (lowest density) to the bottom (highest density).

### 2.3. Western Blotting for VP1 Protein

The VLPs were heated at 95 °C for 10 min before sodium dodecyl sulfate-polyacrylamide gel electrophoresis (SDS-PAGE). Proteins were transferred onto a polyvinylidene difluoride (PVDF; GE Healthcare, Piscataway, NJ, USA) by using a semidry system (Bio-Rad, Hercules, CA, USA). After transfer, the PVDF was incubated in a blocking buffer (5% skim milk in phosphate-buffered saline containing 0.1% Tween-20) at room temperature (rt) for 1 h. The blots were hybridized with VP1 primary antibody (1:1000 in blocking buffer) at rt for 1 h, then washed thrice with PBST. The following secondary antibody (1:10,000 horseradish peroxidase-conjugated goat anti-mouse serum; Dako, Glostrup, Denmark) was incubated with samples at rt for 1 h, and then washed out by PBST. Signals were visualized with Amersham ECL Prime (GE Healthcare, Piscataway, NJ, USA), and recorded with a ChemiDoc MP Imaging System (Bio-Rad, Hercules, CA, USA).

### 2.4. Electron Microscopy for Morphological Study of VP1 Self-Assembled VLP

To examine the size and morphology of VLPs, purified VLPs were layered onto 400-mesh Formvar/Carbon-coated copper grids (EM Sciences, Hatfield, PA, USA). Samples were absorbed on the copper grids for 1 min, then were stained with 4.0% uranyl acetate (Merck KGaA, Darmstadt, Germany). Negatively stained specimens were examined by transmission electron microscope (TEM-HT7700; Hitachi Global, Chiyoda City, Tokyo, Japan).

### 2.5. Matrix-Assisted Laser Desorption Ionization – Time of Flight (MALDI-TOP) MS for Examining Intact VP1 Protein

The VP1 protein (1 μg/μL) was extracted using the solvent mixture chloroform/methanol (2:1 *v/v*, CHCl_3_/MeOH). The matrix solution was 50 mg sinapic acid (SA) matrix in 1 mL acetonitrile/0.1% trifluoroacetic acid (3:7, *v/v*). Samples were prepared by the reversed thin-layer method for MALDI-TOF MS. A 1-μL matrix solution was applied to the top of a vacuum-dried spot, and the sample was added to form a homogenized crystal. The MS target was subsequently introduced into a MALDI-TOF mass spectrometer (Autoflex III; Bruker Daltonics, Bruker Corp., Bremen, Germany).

The sample spot was irradiated with a neodymium:yttrium-aluminum-garnet (Nd: YAG, Nd:Y_3_Al_5_O_12_) laser (355 nm; pulse duration, 3 ns; 200 Hz) for both desorption and ionization. For each sample, an average of 300 laser shots was used to obtain representative mass spectra, which were recorded over the range *m/z* 4000–70,000. All positive-ion mass spectra were acquired in the reflectron mode at an acceleration voltage of 19 kV in pulsed-ion extraction delay mode.

### 2.6. Ultra-Performance Liquid Chromatography (UPLC/MS^E^) Analysis of Peptide Sequences of Digested-VP1 Protein

The VP1 proteins were quantified by data-independent acquisition parallel-fragmentation MS (UPLC/MS^E^) performed with a Waters Xevo G2 Q-TOF mass spectrometer equipped with a nano-electrospray ionization interface (Waters Corporation, Milford, MA, USA) [27]. The VP1 protein (0.1 μg/μL) was dissolved in a solution containing 8M urea (Sigma-Aldrich, Burlington, MA, USA)/50 mM Tris-HCl (pH 8)/5mM Dithiothreitol (Roche Diagnostics Deutschland GmbH, Mannheim, Germany), and incubated at 37 °C for 1 h. The followed procedures were for alkylation and peptide digestion: we added 15 mM iodoacetamide (Sigma-Aldrich, Burlington, MA, USA) to VP1 protein, and incubated it in the dark for 30 min. Then, VP1 protein was digested with Trypsin Gold (Promega, Madison, WI, USA) in 50 mM Tris-HCl (pH 8) buffer, and incubated overnight.

Digested peptides were separated by M-class UPLC (Waters Corporation, Milford, MA, USA), through an M-Class Symmetry C18 Trap Column (130Å, 1.7 µm, Spherical Hybrid, 75 µm × 250 mm) and a BEH C18 column (130Å, 1.7 µm, Spherical Hybrid, 75 µm × 250 mm) under gradient conditions of 300 nL/min flow rate at 40 °C for 70 min. The mobile phase was composed of buffer A (H_2_O + 0.1% formic acid) and buffer B (acetonitrile as the organic modifier and 0.1% formic acid) for molecule protonation. Detailed gradient protocols were initial 1% buffer B, reached 60% at 25th min and 99% at 35th min, maintained 99% at 50th min, and back to 1% at 55th min.

Parallel ion fragmentation was programmed to switch between low (15V) and high (38V) energies in the collision cell. Peptide *m*/*z* signals were collected in the 300–3300 *m*/*z* range. Cone voltage was 40 kV. Glu-fibrinopeptide B (*m*/*z* 785.8426) was used as the real-time lock mass to calibrate data. Collision energies were 0–6V, and cone voltage was 2.65 kV. Lock mass was continuously collected every 10 s in a parallel channel.

### 2.7. UPLC/MS^E^ Data Analysis

The MS data were processed with ProteinLynx Global Server (PLGS, v3.0.3) software (Waters Corporation, Milford, MA, USA). Deisotoped peptide identification and modifications were performed using a database downloaded from UniProt at https://www.uniprot.org (accessed on 5 March 2018). Ion matching requirements were three fragments per peptide, and seven fragments per protein. The post-translational modifications, such as phosphorylation and glycosylation, were analyzed according to the instructional design process of PLGS software [28].

The absolute quantification was performed by comparison of ion intensities to an internal standard of yeast alcohol dehydrogenase (MassPREP ADH digestion standard, SwissProt P00330; Waters Corporation, Milford, MA, USA) which was added to give a final concentration of 10 fmol/mL of on-column sample injection. Quantification was performed with Progenesis QI for proteomics (QIP) software (Nonlinear Dynamics; Waters Corporation, Milford, MA, USA).

### 2.8. Likelihood-Mapping Analysis

For each NoV GI-GVI genogroup from GenBank, three to five complete VP1 sequences were acquired using Blastp (https://blast.ncbi.nlm.nih.gov/Blast.cgi?PAGE=Proteins, accessed on 22 January 2018). Multiple sequence alignment was done using the T-Coffee Multiple Sequence Alignment Program (Comparative Bioinformatics Group, Barcelona, Spain). We manually re-examined the viral sequences and alignment results. Some virus strains were excluded due to insufficient data for the isolation year or location, nonsense or frameshift mutation, or recombination. The final dataset comprised 139 sequences.

For the full-length VP1 and each sub-domain, the best-fit substitution was selected according to the lowest Bayesian information criterion (BIC) score using Molecular Evolutionary Genetics Analysis (MEGA) software (v11; Pennsylvania State University; University Park, PA, USA) [29]. The dataset was quantified by likelihood mapping analysis performed in the TREE-PUZZLE program (v5.3; Heiko A. Schmidt and Arndt von Haeseler, Vienna, Austria) [30]. Analyses of 10,000 quartets were performed using quartet sampling and a neighbor-joining tree with exact parameter estimates. Data were considered reliable for phylogenetic inference if less than 10% of the dots fell in the center of the triangle (unresolved quartets); data were considered unreliable if more than 30% of the dots fell in the unresolved quartets.

### 2.9. Coevolution Analysis

Epistatic interactions constrain evolution; in some key protein sites, mutations are tolerated only after critical compensatory mutations. Therefore, the results of epistasis analysis may facilitate the identification of groups of residues involved in the same function. To understand the function or interaction among detected phosphorylation sites in norovirus, and the co-evolving sites in the VP1 region by the aligned 139 norovirus strains, the Bayesian graphical model (BGM) for co-evolving sites was implemented on the Datamonkey Adaptive Evolution Server (https://www.datamonkey.org/, accessed on 16 August 2021) [31]. A significant association between the two sites was defined as a posterior probability exceeding a default cutoff of 0.5.

## 3. Results

### 3.1. Expression and Characterization of Norovirus VP1 Protein

Ten µL of supernatant from each subfraction from the CsCl ultracentrifuge was collected to measure the refraction index. Buoyant density was determined indirectly: a refractometer was used to obtain the refractive index, which was then converted to buoyant density using the following formula. Table 1 presents the refractive index and density of each subfraction. Notably, subfraction numbers 4, 5, and 6 had densities of 1.3245, 1.3299, and 1.3353 g/cm3, respectively.

Density = 10.8601 × refractive index − 13.4974(1)

**Figure 1:**
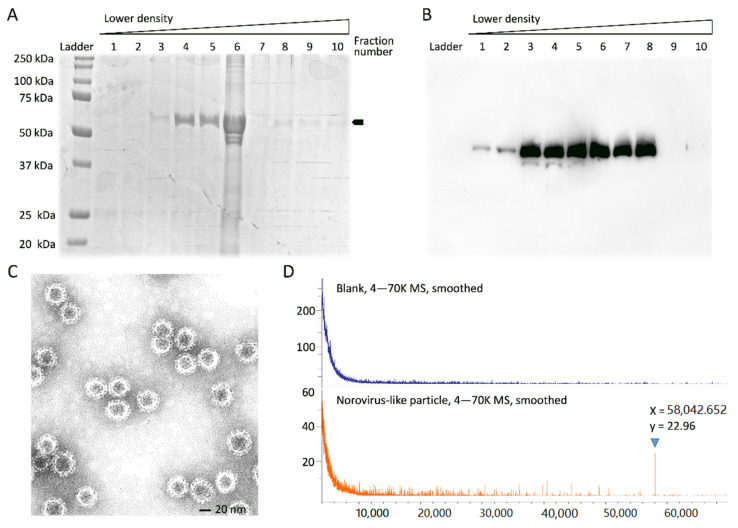
Recombinant norovirus VP1 capsid protein. (**A**) Coomassie blue-stained SDS-PAGE gel for fractions of VP1 proteins; (**B**) Western blot using a polyclonal antibody against VP1 protein; (**C**) Norovirus-like particles under transmission electron microscopy (TEM-HT7700; Hitachi Global); (**D**) MALDI-TOF MS analysis reveals a peak at 58,043 *m/z* (Autoflex III; Bruker Daltonics, Bruker Corp.).

According to the SDS-PAGE results, the 55–60 kDa protein peaked at a density range of 1.3245~3.353 g/cm^3^; the subfraction numbers were 4, 5, and 6 (Figure 1A). Western blot analysis confirmed that VP1 proteins were overexpressed in these subfractions; the range of molecular weights was approximated as 55–60 kDa (Figure 1B).

The VP1 self-assembled particles were visualized by negative staining, and observed by TEM. The norovirus VP1-based VLP was found to be 38 nm (Figure 1C). Next, intact norovirus VP1 proteins of virus strain Hu/GII.4/Saga5/2006/JP were analyzed by MALDI-TOF. A distinct peak was detected at 58,043 *m/z* by 1 µg of VP1 protein (Figure 1D). According to these data, VP1 proteins were successfully produced by the baculovirus expression system with a molecular weight of 58 kDa by western blotting. These VLPs could self-assemble into VLPs (size: 38 nm) by electron microscopy. Further, these VLPs were detectable by MALDI-TOF with a peak at 58,043 *m/z* at a concentration of 17.24 pmol.

### 3.2. VP1 Peptide Sequencing by LC/MS^E^

**Figure 2:**
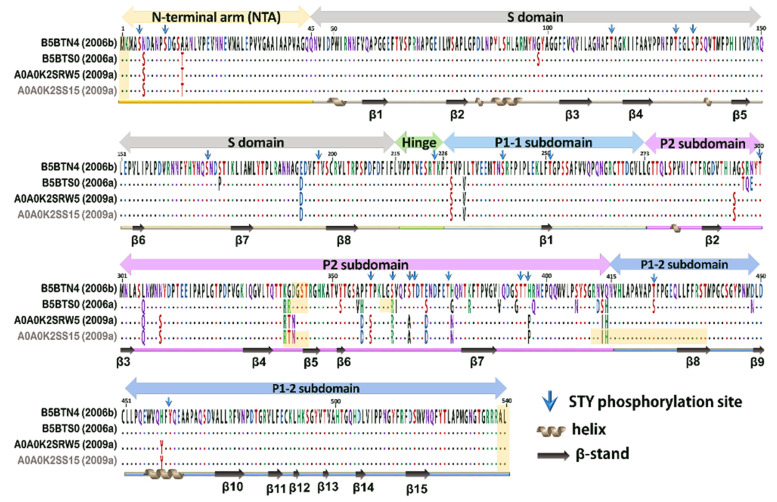
The best-identified norovirus strains. The best-identified strains of variants 2006b and 2006a were B4BTN4 and B5BTS0, respectively. Although the best-identified strain of variant 2009a (A0A0K2SRW5) was A0A0K2SS15, they were identical in amino acid sequence. The virus strains for VLPs were shown in black accession numbers; the best-matched variant 2009a was indicated in the grey accession number. Dots represented amino acids identical to the top sequence-B5BTN4; missing residues were shadowed. The secondary structure of VP1 was noted under the aligned sequences.

Although there were some missing residues, all the genotypes of VLPs were detected correctly. After comparison with reference sequences, these three VLPs were best matched with their original sequences, or with the other strain with an identical VP1 amino acid sequence. In other words, the best-characterized strain (i.e., the sequence with the highest score) of variant 2006b was B5BTN4 (the original strain), with 99.1% (535/540) coverage; variant 2006a was B5BTS0, with 96.7% (522/540) coverage. The best-characterized strain variant 2009a was A0A0K2SS15, with a 94% (505/540) coverage rate; however, A0A0K2SS15 showed 100% similarity to A0A1B1CUK8 (original sequence of the VLP). The coverage rates exceeded 94% at a concentration of 3.6 fmoles. Compared with the secondary structure and functional domain, these common missing residues were located at the N-terminus and C-terminus (two and three missing residues in the N- and C- termini, respectively), and in positions 342–345, this region covers the HBGA-binding targets (343, 344, and 345) (Figure 2). The three tested strains for VLPs were highly similar at the N-terminal arm (NTA), S domain, hinge region, P1-1 subdomain, and P1-2 subdomain, with 2/45, 3/170, 0/10, 2/47, and 3/35 substitutions, respectively. The differences were in the P2 subdomain, particularly at the N-terminal of the P2 subdomain (340–415 residues). These results demonstrated that LC/MS^E^ system has the potential to identify genotypes of norovirus.

### 3.3. Post-Translational Modification Analysis

**Figure 3:**
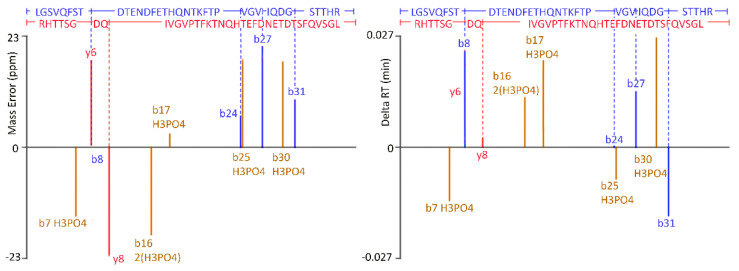
Representative figure of protein phosphorylation. Twenty phosphorylation sites were found. The above figure was 393T and 394T phosphorylation on peptide LGSVQFSTDTEND-FETHQNTKFTPVGVIQDGSTTHR. The peptide sequences and annotated fragment ions were shown in blue (from N-terminal to C-terminal), red (reversed), and brown (phosphorylated). The mass error of the fragment ions was between ± 23 parts per million (ppm), comparing the theoretical *m*/*z* with the experimentally observed *m*/*z*. The Delta retention time (RT) was within ±0.027, reflecting fine reproducibility.

Modifications, such as phosphorylation and glycosylation, may alter the function of viral proteins and interfere with the binding affinity with host cell receptors [31,32]. Thus, we investigated possible post-translational modifications on VLPs by using PLGS software according to the instructional design process. Results revealed that a total of twenty phosphorylation sites can be detected, including positions 5S, 14S, 115T, 130T, 134S, 171S, 197T, 224T, 240S, 251T, 300T, 359T, 364S, 368S, 369T, 377T, 393S, 394T, 425T, and 462Y (Figure 2, marked as ↓). The phosphorylation sites were clustered in the P2 domain and non-β-stand regions. All phosphorylated peptides were convincing, with detectable daughter fragments and high PLGS scores in the software (Figure 3).

### 3.4. Likelihood-Mapping Analysis

The likelihood-mapping analysis is a graphical method to visualize the phylogenetic content of a sequence alignment. Each result is composed of two triangles. One of the two triangles presents dots only. Each dot within the triangle represents the likelihoods of three possible unrooted trees out of 10,000 random quartets. The other triangle divides those dots into seven partitioned regions. The percentage of dots falling in each region is indicated. The three corners of the triangle represent well-resolved phylogeny. The three side-rectangles are net-like regions, hard to distinguish between two of the three topologies. The central triangle area shows a star-like area, indicating “unresolved” [32].

**Figure 4:**
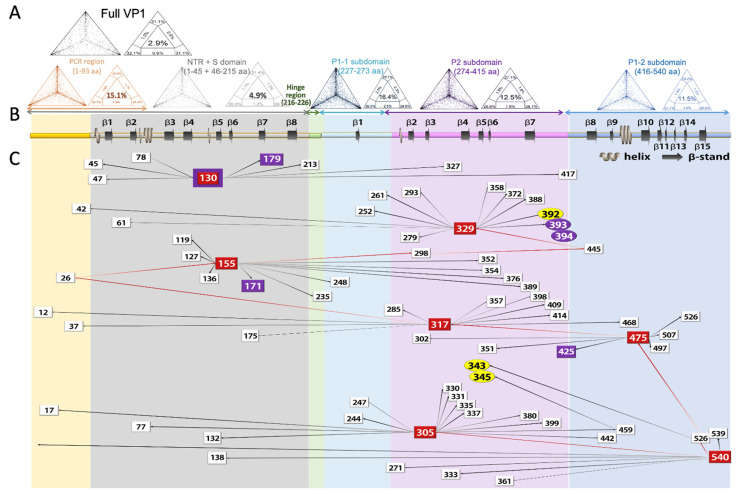
Likelihood mapping, sequence logos, and epistasis analysis of norovirus VP1 sequences. (**A**) Likelihood mapping diagrams of full VP1, S domain, P1-1, P2, and P1-2 domains are shown in the first panel. In the left triangle, each dot represents the likelihoods of three possible unrooted trees out of 10,000 random quartets. The right triangle shows the seven basins of attraction with their corresponding attractors. The numbers indicate the percentage of quartets falling in each region. Numbers in the center area of the triangle correspond to phylogenetic noise; when the number was less than 10%, the data were considered reliable for phylogenetic inference. Likelihood mapping graphics were produced by the TREE-PUZZLE program v5.2. (**B**) The secondary structure guide is located at the third panel (PDB ID code 6ouu). (**C**) Multiple epistatic interactions are shown at the bottom of the figure. Each square represents a residue position that participates in at least one interaction with a marginal posterior probability (PP) exceeding a default cutoff of 0.5. Only epistatic interaction with HBAG binding site (highlighted with yellow cycle), phosphorylation sites (highlighted with purple box), and epistatic interaction group no less than seven (highlighted with red box) are shown. Arrows between squares indicated the epistatic direction between residues, the direction was also enhanced by color level from light to darkness, the PP values are indicated by the line weight. An epistatic network has been shown by the key dominant site position 540.

Results of the likelihood-mapping analysis for (1) full-length VP1, (2) NTR+S domain, (3) P1-1, (4) P2, and (5) P1-2 are shown in (Figure 4A). Full-length aa sequences of the VP1and S domains detected by MS were analyzed by the likelihood-mapping analysis. Results revealed that unresolved quartets were 2.9% and 4.9%, respectively (Figure 4A). Although the N- and C-terminus revealed a few missing residues, they did not interfere with viral identification. In addition, the peptide sequences of P1-1, P2, and P1-2 detected by MS showed that 16.4%, 12.5%, and 11.5% of quartets were unresolved in the likelihood-mapping. Compared to 15.1% by the current PCR-based methodology, the above sequences were superior for identifying noroviruses.

### 3.5. Co-Evalution Analysis

To understand the function or interaction among detected phosphorylation sites in norovirus, epistatic analysis was also performed on 139 norovirus VP1 sequences and 342 pairs of co-evolving sites (*PP* > 0.5). Intriguingly, these 342 pairs revealed numerous multiple epistatic interaction sites (MEISs). Residues with the most MEISs were residues 305, 329, 155, 317, and 540, which had 14, 13, 12, 11, and 8 epistatic interaction pairs, respectively. Additionally, residues 130 and 475 each had seven epistatic interaction pairs (Figure 4, Supplementary Table S1).

Three of the seven MEISs were located in the outermost P2 subdomain (positions 305, 317, and 329), whereas MEIS 540 was located in P1-2. Notably, MEIS 540 had the highest mutation impact, but was not influenced by others. In detail, MEIS 540 showed impacts on both position MEIS 305 (in P2 subdomain) and position 526 (in P1-2). Then, position 526 influenced MEIS 475, MEIS 317, position 26 (in NTA), MEIS 155 residue, residue 298, residue 445, and MEIS 329 (Figure 4B,C, red arrow). Additionally, the interaction network was indirectly or directly associated with HBGA binding site 1 (343 and 345) and site 2 (392 and 393).

Two phosphorylation sites were involved in the epistatic network. The first phos-phorylation site was on 228S, which may have an epistatic interaction with position 343 on the receptor-binding site. The second one was on 78S, showing the mutation impact on MEIS 130.

Collectively, the co-evolution sites identified in VP1 were intimately networked. MEIS 540 (P1-2) was dominant in these interaction chains. Mutation of the MEIS 540 showed great impacts on the multi-epistatic interaction network, especially the receptor binding and antigenic determinant P2 subdomain.

## 4. Discussion

The aim of this mass-based analysis was to establish an easy performed, sensitive, and high-resolution method for laboratory identification of norovirus infection. In this study, the most abundant protein VP1 was produced by a Baculovirus expression system as a standard for the MS-based assay. We demonstrated VLP formation by electron microscopy, which “froze” the protein modification pattern, and revealed the self-assembly ability of the VP1 protein. Limitations of the study were the small number of reference sequences other than GII.4. Further investigations for other genotypes and genogroups are required.

Previously, 57% coverage (concentration range, 0.1 × 10^−12^ to 50 × 10^−12^ mol) has been reported in authentic standards of recombinant VLPs using nanospray tandem MS to detect recombinant HuNoV VP1 protein digests [33]. In the current study, by UPLC/MS^E^ system, 98% coverage and 10 PTM sites achieved unambiguous identification in a concentration of 3.6 × 10^−15^ mol of VLP. The ORF1/ORF2 junction (C region) of the NoV genome serves widely as a target for rapid detection by nucleic acid-based amplification techniques, such as RT-PCR, qRT-PCR, and NASBA [6]. The high mutation rate of NoV results in an inconsistency of genotyping [9]. In the current study, no more than 5% of noise signals (dots on unresolved quartet) were in full VP1 and S domains, which indicated that these regions are sufficient for phylogenetic analysis. Since circulating recombinant strains have been found worldwide, dual typing of ORF1-RdRp (P-type) and ORF 2-VP1 (genotype) is now routinely used for HNoV typing worldwide [6]. Along with the superior coverage rate and sensitivity, UPLC-MS^E^ is a promising tool for NoV typing.

Typical viruses have small genomes that code for less than 20 proteins. Thus, most viral proteins have multiple functions that enable small numbers of viral proteins to hijack the host cell as the machinery for the viral lifecycle. For example, the capsid protein involves decapsidation–encapsidation, trafficking, and modulation of the host immune response [34,35]. Phosphorylation on the flexible regions of the capsid protein, e.g., N- or C-terminus, alters their molecular surface charge and conformation, which then affects how capsids interact with other viral and cellular molecules [36]. This is a dynamic process, like how the discovery of a cluster of differentiation markers in the immune cells relies on cancer cells, especially leukemia cells, to move out of the normal cell cycle, and “freeze” the cell marker in some stage of the carcinogenesis steps. This highlights the potential use of VLPs as tools for understanding the impact of capsid protein phosphorylation on the viral life cycle.

The VP1 protein of norovirus starts with a flexible N-terminal arm (NTA, 1–45 aa), and it has been reported that this area is important for directing capsid assembly [8,37]. The S domain (46–215) exhibits an 8-stranded β-barrel motif, a canonical icosahedral coat building block of all capsid viruses. Two in NTA (5S, 14S), and five phosphorylation S sites (115T, 130T, 134S, 171S, and 197TS) have been detected in this study. Of these, 171S has epistatic interaction by MEIS155, and 179T has epistatic interaction by MEIS130. Besides, 130T itself is a MEIS. This interaction chain suggests phosphorylation 130T might impact the interaction among NTA, S domain, and P2 subdomain. The P domains dimerize to form a protrusion on the capsid surface.

Essentially, the fold of the P1 subdomain (227–273) consists of nine anti-parallel β-strands. The fold of the P2 subdomain (274–416) is a β-barrel of six anti-parallel strands. These β-strands are connected by extensive loops that have varying lengths and are exposed to the surface [8,10]. Analysis of nonsynonymous mutations of HuNoV reveals hypervariability in common structural surface-exposed residues of the P2 domain as a result of immune-driven selection. In contrast, sites corresponding to HBGA-binding targets (343, 344, and 345) are restricted [13,14]. A total of 13 phosphorylation STY sites were found in this region: one in the hinge region (224T); two in P1-1 (240S, 251T); eight in P2 (300T, 359T, 364S, 368S, 369T, 399T, 393T, and 394T); and two in P1-2 (425T and 4625Y). Of these, the 393T and 394T were involved in HBGA-binding, 171S has epistatic interaction by MEIS155, the 179T has epistatic interaction by MEIS130, and notably, the 130T itself is a MEIS. The 425T has epistatic interaction with MEIS475.

The co-evolution analysis in the current study depicted a MEIS axis composed of seven MEISs: two positions in the S domain (130 and 155), three positions in the P2 subdomain (305, 317, and 329), and two positions in the P1-2 subdomain (475 and 540). Position 540 plays the role of a crowbar: mutations at this site impact at least seven other sites. These mutations also have direct or indirect impacts on the HBGA-binding target. Thus, the MEIS interaction axis may have essential roles in triggering conformational changes, and maintaining the balance between receptor binding function and immune escape function.

## 5. Conclusions

This study established a powerful MS-based system (LC/MS^E^) for identifying noroviruses. The system does not require viral genomic amplification or culture. Use of the system in a virological laboratory enables sensitive and efficient detection of mutations, monitoring of genotypes, and identification of protein structural/functional key sites according to VP1 peptide sequencing. By epistatic interaction and phosphorylation analysis, the position 540’s impacts on the co-evolutional network were found, as were one phosphorylated site’s (MEIS130) direct involvement in this MEIS axis, and the possible involvement of two residues’ (393T and 394T) phosphorylation in HBGA-binding.

## Figures and Tables

**Table 1:** Refractive index and density of each subfraction.

Subfraction Number	Refractive Index	CsCl Density (g/cm^3^)
1	1.3626	1.3006
2	1.3633	1.3082
3	1.3639	1.3147
4	1.3648	1.3245
5	1.3653	1.3299
6	1.3658	1.3353
7	1.3666	1.3440
8	1.3667	1.3451
9	1.3679	1.3581
10	1.3689	1.3690
H_2_O	1.3325	0.9737

## Data Availability

The authors confirm that the data supporting the findings of this study are available within the article [and/or] its supplementary materials.

## Supplementary Materials

The following are available online at https://www.mdpi.com/article/10.3390/v13112332/s1, Table S1: Co-Evolving Pairs of Sites Detected by Bayesian graphical model.
