# Supplementary material for: Mass Spectrometry-Based System for Identifying and Typing Norovirus Major Capsid Protein VP1
Source: Viruses. 2021 Nov 22;13(11):2332. doi: 10.3390/v13112332 (PMC8624548; doi:10.3390/v13112332)
Supplement: Supplementary file 1 [file viruses-13-02332-s001.zip › viruses-1414264-supplementary.pdf]

Supplementary Table S1: Co-Evolving Pairs of Sites Detected by Bayesian graphical model.

Site 1	Site 2	P [Site 1 –> Site 2]	P [Site 2 –> Site 1]	P [Site 1 <–> Site 2]
2	100	0	0.96	0.96
6	540	0	0.99	0.99
9	138	0	1	1
10	115	0.91	0	0.91
11	422	0.97	0	0.97
12	71	1	0	1
12	317	0.001	0.84	0.84
13	14	0.43	0.57	1
14	223	0.92	0.02	0.94
14	308	0.76	0	0.76
15	21	0	0.96	0.96
15	467	1	0	1
16	200	0.001	0.93	0.93
17	305	0	0.99	0.99
17	316	0.99	0	0.99
21	224	0.98	0.009	0.99
21	239	0.98	0.006	0.99
21	287	0.93	0	0.93
21	288	0.73	0	0.73
21	436	0.002	0.97	0.97
22	437	0	0.99	0.99
23	362	0.01	0.99	1
24	27	0	1	1
25	134	1	0	1
25	436	1	0	1
26	155	1	0	1
26	317	0	0.99	0.99
26	471	1	0	1
27	121	0	0.96	0.96
27	170	0.98	0	0.98
27	369	0.97	0	0.97
28	514	0	0.99	0.99
30	152	0.7	0	0.7
33	150	0	0.94	0.94
35	192	0.9	0	0.9
36	368	0	0.6	0.6
37	317	0	0.78	0.78
39	99	0	0.98	0.98
39	363	1	0	1
42	329	0	0.87	0.87

Site 1	Site 2	P [Site 1 –> Site 2]	P [Site 2 –> Site 1]	P [Site 1 <–> Site 2]
45	130	0	1	1
45	164	0.96	0	0.96
45	377	0.99	0	0.99
45	431	0.86	0	0.86
47	130	0	1	1
47	346	0.8	0	0.8
53	121	0	0.98	0.98
53	211	0.97	0	0.97
53	314	0.62	0	0.62
53	382	0.95	0	0.95
53	523	1	0	1
54	120	0.99	0.01	1
54	257	0	0.99	0.99
54	315	0.54	0	0.54
56	198	0	0.94	0.94
59	220	0.72	0.28	1
61	214	0.88	0	0.88
61	238	1	0	1
61	295	0.82	0	0.82
61	329	0	0.57	0.57
66	205	0.73	0	0.73
69	106	0.079	0.92	1
69	379	1	0	1
71	255	0.99	0	0.99
74	189	0.32	0.36	0.68
74	487	0.33	0.28	0.61
75	350	0	0.56	0.56
77	305	0	0.99	0.99
77	489	1	0	1
78	80	0.57	0	0.57
78	99	0.001	0.69	0.69
78	130	0.59	0.081	0.68
78	246	0.95	0	0.95
78	493	1	0	1
79	177	0.88	0.12	1
79	397	0.83	0.01	0.84
82	139	0.47	0.51	0.98
84	156	0.99	0	0.99
84	335	0	0.92	0.92
86	537	0.36	0.41	0.77
90	271	0	1	1
93	150	0.001	0.51	0.52

Site 1	Site 2	P [Site 1 –> Site 2]	P [Site 2 –> Site 1]	P [Site 1 <–> Site 2]
94	457	0	0.95	0.95
98	181	0.87	0.015	0.89
98	260	0.82	0	0.82
98	403	0	0.9	0.9
99	518	1	0	1
100	312	0.94	0	0.94
103	249	0	0.97	0.97
104	193	1	0	1
104	528	0	0.86	0.86
106	402	0	0.78	0.78
107	355	0	0.91	0.91
108	273	0.6	0	0.6
108	494	0.9	0	0.9
108	501	0	1	1
119	155	0	0.95	0.95
120	178	0.58	0	0.58
121	195	0.99	0	0.99
121	236	0.55	0	0.55
121	492	0.64	0.32	0.96
122	129	0	0.62	0.62
124	382	0	0.75	0.75
127	155	0	1	1
127	311	0.73	0	0.73
129	415	0.96	0.008	0.97
130	179	0.83	0	0.83
130	213	0.94	0	0.94
130	327	0.81	0	0.81
130	417	1	0	1
131	499	0.95	0	0.95
132	144	1	0	1
132	305	0	0.98	0.98
135	457	0	0.94	0.94
136	155	0	1	1
136	373	0.79	0	0.79
137	451	0.26	0.3	0.56
138	416	0.99	0	0.99
138	540	0	1	1
140	225	0	1	1
141	279	0	1	1
141	324	0.98	0	0.98
141	519	0.79	0	0.79
145	239	0.005	0.98	0.99

Site 1	Site 2	P [Site 1 –> Site 2]	P [Site 2 –> Site 1]	P [Site 1 <–> Site 2]
146	352	0	0.87	0.87
150	173	0	0.9	0.9
155	171	0.96	0	0.96
155	235	1	0	1
155	248	1	0	1
155	298	1	0	1
155	352	1	0	1
155	354	0.83	0	0.83
155	376	1	0	1
155	389	1	0	1
161	320	0.72	0	0.72
161	519	0.19	0.81	1
163	202	1	0	1
163	403	0.92	0.016	0.93
163	464	0	0.98	0.98
163	480	0.77	0	0.77
164	502	0.92	0	0.92
165	323	0.89	0	0.89
165	533	0	0.93	0.93
166	337	0	0.99	0.99
168	348	0	0.87	0.87
169	267	0.37	0.21	0.58
173	341	0.72	0	0.72
173	437	0	0.59	0.59
174	250	0	0.81	0.81
175	317	0	0.59	0.59
176	319	1	0	1
176	449	0	0.92	0.92
180	284	0.74	0	0.74
180	492	0	0.64	0.64
188	411	0.82	0	0.82
188	412	0.73	0	0.73
188	526	0	0.88	0.88
189	487	0.42	0.27	0.68
190	493	0.002	0.97	0.97
191	255	0	0.71	0.71
191	268	0.68	0	0.68
195	232	0.57	0	0.57
195	443	0.53	0	0.53
195	462	0.52	0	0.52
198	422	0	0.51	0.51
199	427	0.29	0.32	0.61

Site 1	Site 2	P [Site 1 -> Site 2]	P [Site 2 -> Site 1]	P [Site 1 <-> Site 2]
199	476	0.19	0.48	0.67
200	277	0.99	0	0.99
200	492	0	0.71	0.71
203	491	0.003	0.76	0.76
207	369	0	0.99	0.99
208	443	0	0.54	0.54
213	470	0.91	0	0.91
216	480	0	1	1
219	369	0	0.51	0.51
226	530	0	0.55	0.55
227	452	0	0.96	0.96
228	343	0	1	1
229	489	0	0.86	0.86
233	321	0.98	0	0.98
233	530	0.086	0.91	0.99
234	357	0	0.97	0.97
236	332	0.51	0.007	0.52
237	293	0	0.98	0.98
244	305	0	0.99	0.99
245	469	0	1	1
247	305	0.19	0.61	0.8
247	509	0.38	0.14	0.52
248	496	0.6	0	0.6
249	257	0.01	0.99	1
251	336	0.36	0.62	0.98
252	329	0	1	1
253	313	0	0.68	0.68
254	493	0	0.96	0.96
256	289	0.79	0	0.79
256	366	1	0	1
256	404	0.68	0	0.68
256	437	0.011	0.81	0.83
257	261	0	0.65	0.65
257	310	1	0	1
257	505	1	0	1
258	285	0	1	1
260	455	0.44	0.17	0.61
261	329	0	1	1
266	365	0	0.94	0.94
271	400	0.99	0	0.99
271	534	0.97	0.03	1
271	540	0.09	0.79	0.88

Site 1	Site 2	P [Site 1 –> Site 2]	P [Site 2 –> Site 1]	P [Site 1 <–> Site 2]
272	428	0.23	0.75	0.98
278	514	1	0	1
279	329	0	1	1
280	381	0	0.6	0.6
281	448	0	0.99	0.99
282	441	0	0.78	0.78
285	317	0	0.95	0.95
286	391	0	0.54	0.54
288	466	0.89	0.11	1
289	329	0.35	0.18	0.53
290	366	0	0.5	0.5
291	446	0	0.7	0.7
292	438	0	1	1
293	329	0	0.75	0.75
294	388	0	1	1
297	437	0.43	0.53	0.97
298	445	0.72	0.001	0.72
299	465	0	1	1
300	507	0	0.89	0.89
301	381	0	1	1
301	501	0.93	0	0.93
302	475	0	0.83	0.83
303	388	0	0.94	0.94
304	438	0	0.98	0.98
305	330	0.88	0	0.88
305	331	0.99	0	0.99
305	335	0.99	0	0.99
305	337	0.99	0.01	1
305	380	0.99	0	0.99
305	399	0.98	0	0.98
305	442	0.99	0	0.99
305	459	0.99	0	0.99
305	540	0	0.79	0.79
306	330	0	0.75	0.75
307	511	0	0.86	0.86
309	413	0.015	0.95	0.97
313	328	1	0	1
313	385	0.77	0	0.77
313	402	0	0.54	0.54
313	483	0.92	0	0.92
317	357	0.56	0	0.56
317	398	0.72	0	0.72

Site 1	Site 2	P [Site 1 –> Site 2]	P [Site 2 –> Site 1]	P [Site 1 <–> Site 2]
317	409	1	0	1
317	414	0.99	0	0.99
317	468	0.99	0	0.99
317	475	0	1	1
324	486	0.98	0.003	0.98
329	358	0.66	0	0.66
329	372	1	0	1
329	388	1	0	1
329	392	0.9	0	0.9
329	393	1	0	1
329	445	0	0.64	0.64
332	383	0.47	0.26	0.73
332	516	0.39	0.23	0.62
333	512	0.63	0	0.63
333	540	0	1	1
334	348	0	0.89	0.89
336	430	0.54	0	0.54
336	457	0.52	0	0.52
337	370	0.88	0	0.88
338	370	0	0.54	0.54
339	377	0	0.62	0.62
340	377	0	0.98	0.98
341	453	0.83	0	0.83
343	526	0	0.99	0.99
344	370	0	0.67	0.67
344	375	0.67	0.33	1
345	347	1	0	1
345	459	0	1	1
348	370	0.06	0.94	1
348	441	0.87	0.06	0.93
350	368	0	1	1
351	475	0	1	1
353	423	0	0.98	0.98
355	357	0	0.99	0.99
356	401	0.54	0	0.54
356	437	0	1	1
356	504	1	0	1
361	384	0.55	0.001	0.55
361	463	0.6	0.39	0.99
361	540	0.19	0.35	0.54
362	403	0.001	0.86	0.86
363	428	0.61	0.002	0.61

Site 1	Site 2	P [Site 1 –> Site 2]	P [Site 2 –> Site 1]	P [Site 1 <–> Site 2]
364	445	0	0.57	0.57
367	369	0	0.99	0.99
368	445	0.28	0.7	0.98
369	446	1	0	1
370	371	1	0	1
372	478	1	0	1
375	391	1	0	1
376	444	0.94	0	0.94
378	465	0	1	1
381	430	0	0.73	0.73
381	485	0.98	0	0.98
383	516	0.29	0.34	0.64
386	457	0	1	1
387	472	0	0.91	0.91
389	390	0.98	0	0.98
402	435	0.021	0.61	0.64
403	525	0.86	0	0.86
407	437	0	0.81	0.81
412	506	1	0	1
413	465	0	0.99	0.99
421	440	0	0.93	0.93
422	452	0.61	0.029	0.64
425	475	0	1	1
426	462	0.12	0.82	0.95
427	476	0.32	0.4	0.72
430	469	0.83	0	0.83
430	503	1	0	1
435	450	0.69	0.31	1
435	528	0.59	0	0.59
438	526	0	0.96	0.96
439	473	0.048	0.45	0.5
439	517	0.016	0.51	0.53
440	458	0.99	0	0.99
440	507	0	0.99	0.99
449	493	0	0.95	0.95
457	464	0.88	0	0.88
465	509	0.079	0.79	0.87
473	517	0.53	0.43	0.97
475	497	0.97	0	0.97
475	507	0.9	0	0.9
475	526	0.03	0.97	1
481	512	0	0.55	0.55

Site 1	Site 2	P [Site 1 –> Site 2]	P [Site 2 –> Site 1]	P [Site 1 <–> Site 2]
492	522	0.92	0	0.92
495	515	0.46	0.29	0.75
500	533	0	0.91	0.91
508	523	0	0.84	0.84
509	530	0.52	0.47	0.99
526	540	0	0.65	0.65
535	536	0.46	0.54	1
539	540	0	0.98	0.98

Columns site 1 and site 2 show the sites determined to have at least one co-evolutionary interaction. Protein regions: N-terminus arm (a.a. 1-45) colored in light orange; shell domain (a.a. 46-215) in light grey; hinge region (a.a. 216-226) in light green; P1-1 (a.a. 227-273) and P1-2 subdomain (a.a. 416-5840) in light and darker blue; P2 subdomain (a.a. 247-415) in light purple. Column P [Site 1 –> Site 2], P [Site 2 –> Site 1], and P [Site 1 <–> Site 2] shows the posterior probability (PP) value of site 1 and site 2 influence with each other, the arrow between site 1 and site 2 indicates the influence direction. The background colored gradient by posterior probability values. This coevolution analysis was analyzed by the spidermonkey/BGM implemented in datamonkey website (http://www.datamonkey.org/bgm).
